# Mapping the disparities in childhood immunization status and determinants in East Africa using ordinal logistic regression analysis

**DOI:** 10.1038/s41598-025-28853-2

**Published:** 2025-12-29

**Authors:** Demeke Lakew Workie, Aster Addisu Dires, Abay Kassa Teklie, Tadele Kassahun Wudu

**Affiliations:** 1 Department of statistics, Bahir Dar University, Bahir Dar, Ethiopia; 2 Department of statistics, Debark University, Debark, Ethiopia

**Keywords:** Childhood immunization status, East africa, Mapping, Ordinal logistic regression, Spatial analysis, Paediatrics, Paediatric research

## Abstract

East Africa, a region where vaccine-preventable diseases are prevalent, has faced significant challenges in immunization services despite the global success of the Expanded Programme on Immunization in reducing childhood mortality. This study utilized National Demographic and Health Survey (DHS) data from 10 countries, involving 22,734 participants aged 12 to 23 months, to analyze the geographic distribution of child immunization and influencing factors. Employing the kriging interpolation technique, we mapped disparities across 132 regions, revealing that 25% reported less than 67% of children fully immunized, with nearly half achieving only 48% coverage. Key factors affecting immunization rates included parental education, household characteristics, media access, birth intervals, and health documentation. Educated families demonstrated higher vaccination rates, while barriers faced by female-headed households were notable. The findings underscore the need for targeted policies and region-specific strategies to enhance immunization and public health efforts in East Africa.

## Introduction

In an effort to significantly lower the burden of illness and death brought on by the main infectious diseases (like diphtheria, tetanus, and polio) affecting infants and children, the World Health Organization (WHO) established the Expanded Programme on Immunization (EPI) in 1974. As a result, during the past half-century, the EPI has saved 154 million lives, reduced infant mortality by almost 40%, and avoided millions from being crippled^1–3^. Also, it has led to the complete elimination of smallpox and a significant decrease of 94% in global cases of polio and neonatal tetanus^4,5^. Immunization boosts the immune system and enables the body to use its natural defenses to combat specific illnesses^6^. It is a highly effective and affordable health intervention technique, preventing over 4 million deaths each year. Despite this progress, across the globe the number of zero-dose children (i.e., children who have not received the first dose of the diphtheria-tetanus-pertussis (DTP) vaccine) rose from 12.8 million in 2019 to 14.5 million in 2023, reversing prior progress^7^.

Global immunization efforts have stalled or reversed in many regions. Between 2000 and 2019, it has prevented an estimated 37 million deaths in 98 low- and middle-income countries (LMICs), resulting in a 45% reduction in mortality from vaccine-preventable diseases^8^. However, DTP3 (a key indicator of immunization coverage) declined between 2019 and 2023, dropping by 7% points (from 75% to 68%) in low-income countries, while high-income nations recovered to near pre-2019 levels. These global statistics mask significant disparities, with low-income countries lagging behind higher-income nations. Pandemic-related disruptions to routine childhood vaccination are expected to amplify the existing gaps, further hindering countries from achieving global immunization targets^1^.

Each year, around 3 million children in the African region die from infectious diseases. Many of these deaths are preventable with immunization, yet an estimated 20% of children in the region do not receive the necessary vaccines^9,10^. Sub-Saharan Africa continues to have the highest child mortality rate worldwide, averaging 76 deaths per 1,000 live births in 2019. More than 80% of the 5.2 million child deaths occurred in sub-Saharan Africa, Central and Southern Asia^9^. East Africa still has a high rate of incomplete childhood vaccination, which emphasizes the need for more study to improve and prioritize vaccination campaigns for every child’s health and well-being^11^. Although low rates of basic childhood vaccination in east Africa which range from 39.5% in Ethiopia to 85% in Burundi, below the national target of at least 90% (80% coverage in each district) and the WHO goal of ≥ 90% remain a serious public health threat^12,13^.

In East Africa, some studies have investigated factors associated with childhood immunization status using classical models such as binary logistic regression. While binary logistic regression is effective in analyzing immunization status, it does not provide sufficient information to study patterns across different levels of immunization^11,14^. To address these gaps, we employed an ordinal logistic regression model to examine the patterns of immunization levels among children. Health indicators give policymakers access to data at the lowest administrative levels—continental, national, sub-national, or any other lower level^15^. Hence, according to what we have found so far to the best of our studies, there is no statistical and spatial analysis that provides sufficient data to establish the impacts of national diversity on the vaccination status of children in East Africa. So to eliminate child mortality and disability caused by vaccine-preventable diseases, evidence at both regional and national levels is essential. Such evidence supports the planning of targeted activities tailored to the socio-demographic context and the backgrounds of children within each society, allowing for regional-level customization in every nation. Therefore, this study aimed to map the distributions in the prevalence of childhood immunization status within and between East African (EA) countries and to investigate the associated factors among children aged 12–23 months using data from the most recent Demographic and Health Surveys conducted in 10 countries.

## Methods and analysis

### Study area and data

The African continent was divided into five regions by the United Nations (UN) Statistics Division and among these regions, East Africa is the largest as it covering 19 countries (Burundi, Comoros, Djibouti, Ethiopia, Eritrea, Kenya, Madagascar, Malawi, Mauritius, Mozambique, Reunion, Rwanda, Seychelles, Somalia, Somaliland, Tanzania, Uganda, Zambia, and Zimbabwe.)^16,17^. The population of Eastern Africa accounted for 6.13% of the global population and ranked as the most populous sub region in Africa^18^. The child population in the region had increased nearly sevenfold since 1950.

**Fig. 1:**
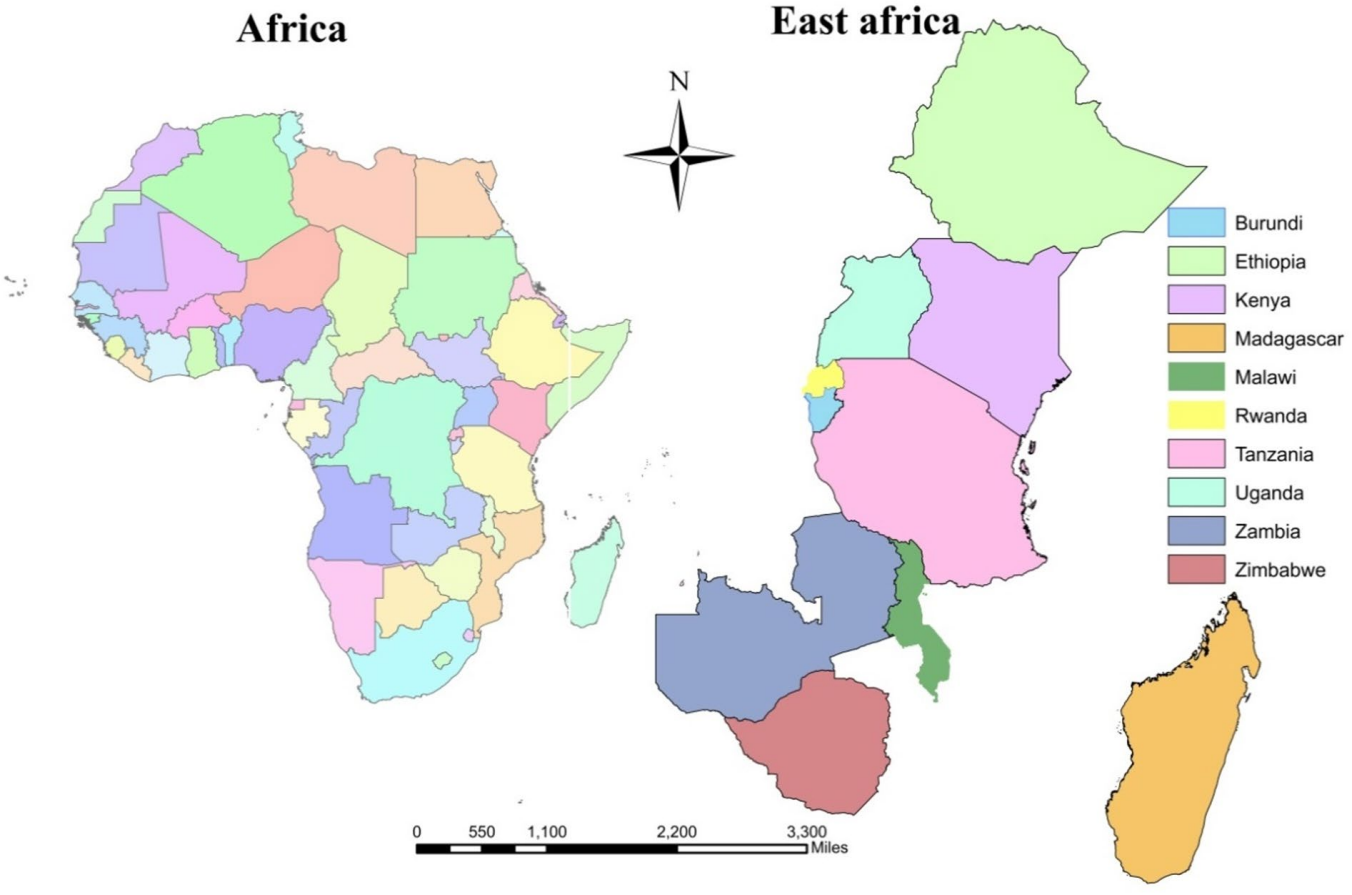
Maps of Africa and the selected East African countries.

**Fig. 2:**
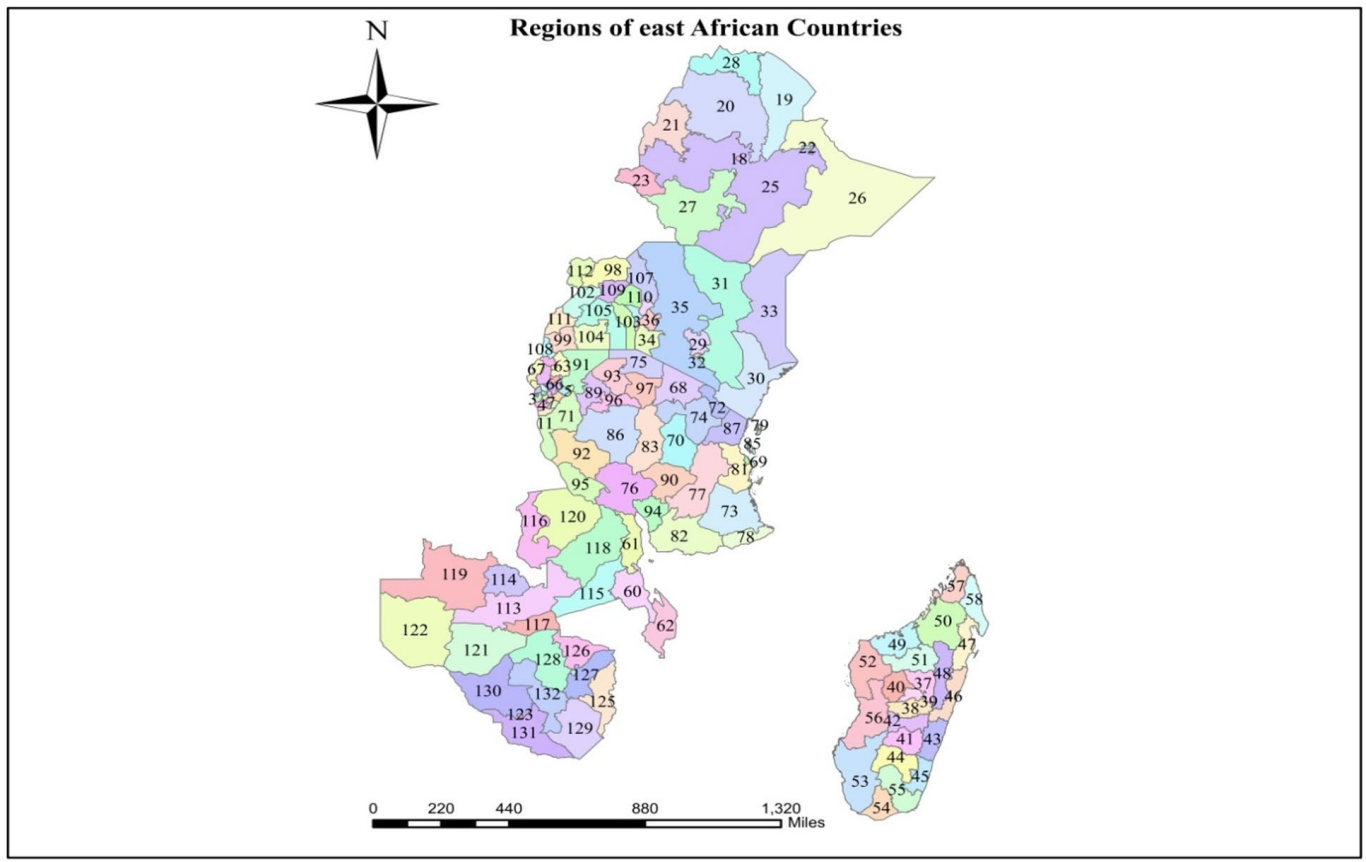
Description of 132 regions of East African Countries.

Data for this study were obtained from the recent Demographic and Health Surveys (DHS) in 10 East African (EA) countries (Fig. 1). The choice of the 10 countries from EA was based on the availability of the variables of interest for which the GPS coordinates (latitude and longitude) of household clusters were available. The DHS served the source of the country-level data with most recent surveys, and the studies were conducted between 2015 and 2022. We used DHS data because it is the largest source of data for low- and middle-income countries. A total of 22,734 children in EA were included in the study. More generally, the Kids Recode files of the DHS were used. The DHS is a nationwide survey conducted in LMICs every five years. It is representative of each country and targets core maternal and child health indicators, such as child immunization status. Multistage sampling was used to select the sample for each survey in various countries. Hence, the first stage of the sampling procedure involved the selection of clusters (enumeration areas (EAs)), which was followed by systematic household sampling within the selected EAs. The administrative shape files were obtained from free global administrative unit databases provided by the DIVA-GIS project (http://www.diva-gis.org)^19^. For administrative purposes, the country is divided into regions, consisting of a total of 132 administrative areas/regions/provinces, which serve as the basis for the entire analysis (Fig. 2).

Description of 132 regions of East African Countries.

**Table:** 

Code	Region Name	Code	Region Name	Code	Region Name
1	Bubanza	45	Atsimo atsinanana	89	Geita
2	Bujumbura mairie	46	Atsinanana	90	Iringa
3	Bujumbura rural	47	Analanjirofo	91	Kagera
4	Bururi	48	Alaotra mangoro	92	Katavi
5	Cankuzo	49	Boeny	93	Mwanza
6	Cibitoke	50	Sofia	94	Njombe
7	Gitega	51	Betsiboka	95	Rukwa
8	Karusi	52	Melaky	96	Shinyanga
9	Kayanza	53	Atsimo	97	Simiyu
10	Kirundo	54	Androy	98	Acholi
11	Makamba	55	Anosy	99	Ankole
12	Muramvya	56	Menabe	100	Bugishu
13	Muyinga	57	Diana	101	Bukedi
14	Mwaro	58	Sava	102	Bunyoro
15	Ngozi	59	Antananarivo	103	Busoga
16	Rutana	60	Central Malawi	104	South Buganda
17	Ruyingi	61	Northern Malawi	105	North Buganda
18	Addis Ababa	62	Southern Malawi	106	Kampala
19	Afar	63	Eastern/iburasirazuba	107	Karamajo
20	Amhara	64	Kigali city/umujyi wa Kigali	108	Kigezi
21	Benshangul gumuz	65	Northern amajyaruguru	109	Lango
22	Dire Dawa	66	Southern amajyepfo	110	Teso
23	Gambella	67	Western/iburengerazuba	111	Tooro
24	Harari	68	Arusha	112	West nile
25	Oromia	69	Dar es salaam	113	Central Zambia
26	Somali	70	Dodoma	114	Copperbelt
27	SNNPR	71	Kigoma	115	Eastern Zambia
28	Tigray	72	Kilimanjaro	116	Luapula
29	Central Kenya	73	Lindi	117	Lusaka
30	Coast	74	Manyara	118	Muchinga
31	eastern Kenya	75	Mara	119	North western Zambia
32	Nairobi	76	Mbeya	120	Northern Zambia
33	North eastern Kenya	77	Morogoro	121	Southern Zambia
34	Nyanza	78	Mtwara	122	Western Zambia
35	Rift vally	79	Kaskazini pemba	123	Bulawayo
36	Western Kenya	80	Kaskazini unguja	124	Harare
37	Analamanga	81	Pwani	125	Manicaland
38	Vakinankaratra	82	Ruvuma	126	Mashonaland central
39	Itasy	83	Singida	127	Mashonaland east
40	Bongolava	84	Kusini pemba	128	Mashonaland west
41	Haute matsiatra	85	Kusini unguja	129	Masvingo
42	Anamoron’i mania	86	Tabora	130	Matabeleland north
43	Vatovavy fitovinany	87	Tanga	131	Matabeleland south
44	Ihorombe	88	Mjini Magharibi	132	midlands

### Variables of the study

In this study, regions were used as the spatial units of analysis^20^. The 10 East African countries are divided into 132 administrative areas (regions).

### Outcome variable

The dependent variable was the immunization status of children aged 12–23 months. As recommended by the World Health Organization (WHO), basic childhood vaccinations include polio, pentavalent (DPT plus Haemophilus influenzae, and hepatitis B), measles, and Bacillus Calmette-Guérin (BCG) vaccines, which prevent common childhood infections^6,9,21,22^. A child was classified as “fully immunized” if they had received all the vaccines listed above in accordance with WHO EPI guidelines. Children who had received at least one, but not all, of the recommended doses were classified as “partially immunized.” Those who had not received any of these vaccines were categorized as “not immunized”^6^.

### Independent variables

**Fig. 3:**
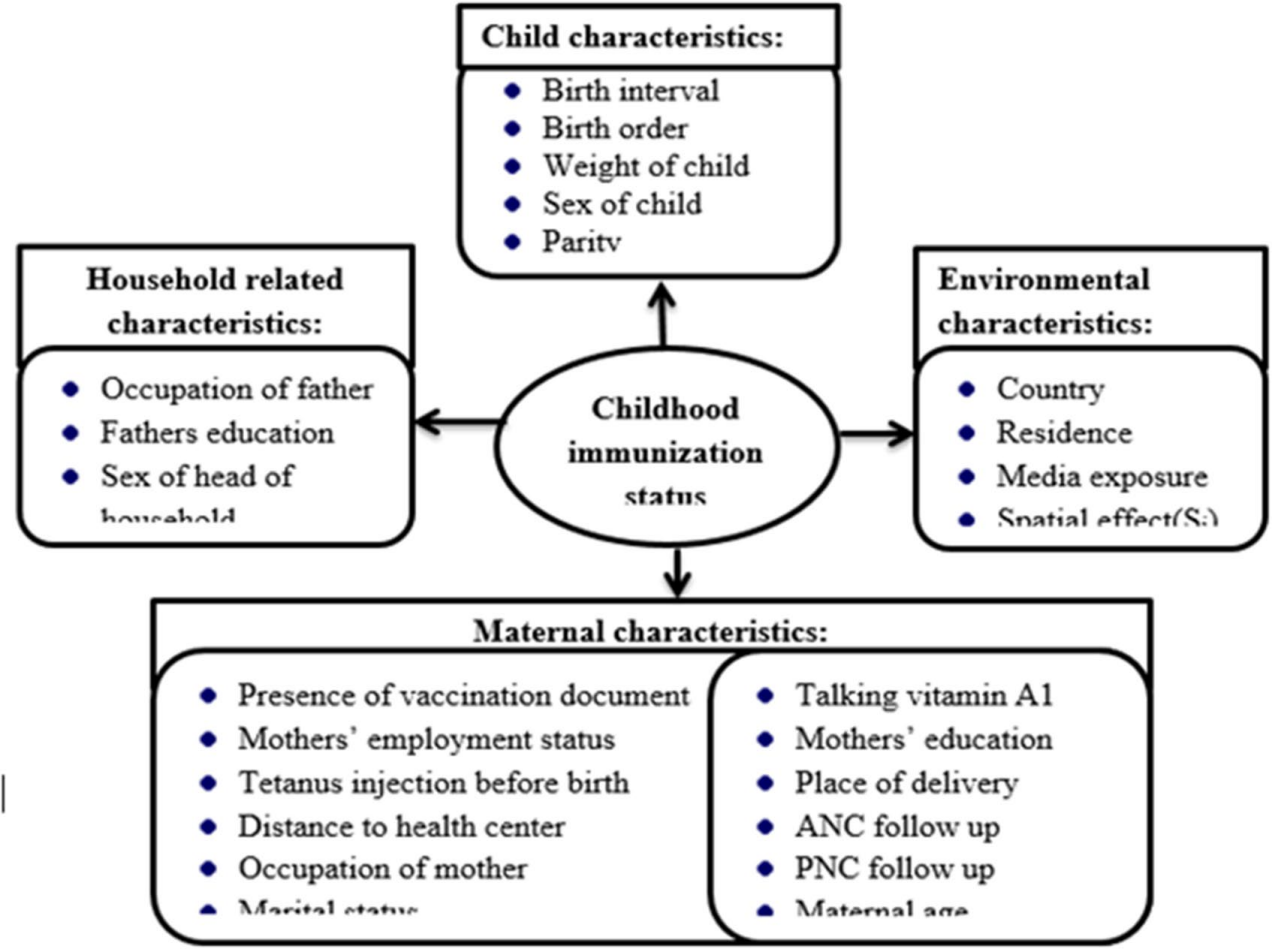
Conceptual framework for features description.

The independent variables included factors related to the child, mother, and household characteristics. These variables were selected based on evidence from the literature^14,22–28^ (Fig. 3).

### Data analysis

The outcome variable was coded as an ordinal variable with three categories: “not-immunized” (0), “partially immunized” (1), and “fully immunized” (2). Data analysis was conducted using STATA 17 and ArcGIS version 10.8 was utilized for mapping purposes. The analysis consisted of two key stages. In the first stage, descriptive statistics, including frequencies, percentages, and means were calculated. This stage also included spatial interpolation techniques, hot spot analysis, and the visualization of immunization status distributions across different categories using pie charts. In the second stage, ordinal logistic regression analysis was performed to identify factors associated with the immunization status of children. As missing data are commonly encountered in DHS data, the multiple imputations technique was used to handle this issue^29^.

### Ordinal logistic regression

Ordinal logistic regression was used to analyze the relationship between an ordinal dependent variable and a set of independent variables^30,31^. Several types of ordinal logistic regression models exist. The most commonly used models are the proportional odds model (POM)^32,33^, partial proportional odds model (PPOM)^34^, adjacent category logit model (ACLM)^33^, continuation ratio logit model (CRLM)^33^ and generalized ordered logit model (GOLM)^35^. For this study, the partial proportional odds model (PPOM)^33^ was employed to determine the most appropriate model for the data, with the final model selection guided by statistical tests.

### Partial proportional odds model (PPOM)

The Partial Proportional Odds Model (PPOM) was selected for its flexibility, as it allows the proportional odds assumption to be relaxed for a subset of explanatory variables. It models variables that meet the assumption with a single coefficient (β), while variables that violate the assumption are modeled with category-specific coefficients (${\delta _j}$)^34^. This allows for variations in the effect of certain predictors across different category comparisons of the outcome variable. The model was specified as:

1 $$\:logit\left({\gamma\:}_{j}\right)=log\left(\frac{{\gamma\:}_{j}}{1-{\gamma\:}_{j}}\right)={\eta\:}_{i}={\theta\:}_{j}+{{\mathbf{x}}_{\mathbf{i}}}^{{\prime\:}}\beta\:+\mathbf{q}{\delta\:}_{j}$$

Where: $\:{\theta\:}_{j}$ is the threshold/cut point, **x** is a vector containing the full set of independent variables not violating parallel line assumption,and **q** is a vector of a subset of independent variables violating parallel line assumption; β and $\:\delta \:$ are the regression coefficients of those predictors, respectively.

### Spatial analysis

Traditional ordinal logistic regression models fail to account for the dependency of spatially collected data on its geographic location^36^. Observed values were not truly independent, as spatial proximity can introduce correlations. Neglecting these spatial effects in the model leads to biased estimations^37^. To address this issue, a spatial weight matrix (W) was incorporated to adjust relationships between dependent variables, independent variables, and residual terms, capturing spatial interactions more accurately. The spatial weight matrix (**W**) was a square symmetric matrix with elements equal to one if units i and j are neighbors of one another and zero otherwise^20^. By convention, the self-neighbor relation is excluded, so that the diagonal elements of **W** are zero, Wij = 0.

For the spatial autocorrelation and hot-spot analysis, we treated the data as regional counts. The number of children in region **i** (**i** = 1, 2,., 132) who were not, partially, or fully immunized was denoted as x_i_. For the purpose of these spatial statistics, which are designed for count data, the value x_i_ was assumed to follow a Poisson distribution^38^.

### Spatial weights matrix

The spatial weight matrix (**W**) was a square symmetric $\:\mathrm{n}\times\:\mathrm{n}$ matrix with elements equal to one if units i and j are neighbors of one another and zero otherwise. By convention, the self-neighbor relation is excluded, so that the diagonal elements of **W** are zero, W_ij_ = 0, for i = j^39,40^.

2 $$\begin{aligned}\mathbf{W}& =\left[\begin{array}{cccc} 0& \:{w}_{12} &\:\dots\: &\:{w}_{1n}\\ \:{w}_{21} &\:0 &\:\dots\: &\:{w}_{2n}\\ \vdots &\vdots &\:\ddots\:& \vdots \\\:{w}_{n1}&\:{w}_{n2}&\:\dots\:&\:0\end{array}\right], \\ {w}_{ij} &=\left\{\begin{array}{c}1\\ \:0 \end{array}\right.\begin{array}{c}if\:unit\:i\:and\:j\:are\:contiguous\\\:other\:wise\end{array}\end{aligned}$$

Assuming the weights are binary, the most common methods for constructing such a. matrix are as follows^41^.

- Rook case contiguity: Two regions are spatial neighbors if they share a common border (on any side).
- Bishop case contiguity: two spatial regions meet at a point. This is similar to two elements of a graph meeting at a vertex.
- Queen’s case (used in this study): It is the combination of Rook’s and Bishop’s case.

Since, W is a square matrix of dimensions$\:n\times\:n$, it results in a $\:132\times\:132$ square matrix containing 0 and 1 in the case of this study. This matrix is then multiplied by a 1 × 132 matrix representing of the countries’ proportion ($\:{y}_{i}$). Finally, dividing the result by the respective number of neighbors for each countries yields the auto-covariate term.

### Spatial autocorrelation analysis

It measures the degree to which a phenomenon of interest was correlated to itself in space. Tobler stated that “everything is related to everything else, but near things are more related than distant things.”^37^. The presence of spatial autocorrelation and the appropriate weight matrix (W) in the dataset was evaluated by using Moran’s I^42–44^. Moran’s I assigns a weight (Wij) to each pair^20^, quantifying the spatial pattern. The test is defined as follows:

3 $$\:I=\frac{n}{{h}_{0}}\left(\frac{\sum\:_{i=1}^{n}\sum\:_{j=1}^{n}{w}_{ij}({x}_{i}-\stackrel{-}{x})({x}_{j}-\stackrel{-}{x})}{\sum\:_{i=1}^{n}({{x}_{i}-\stackrel{-}{x})}^{2}}\right)$$

Where n is the number of points under study; $\:{x}_{i}$, $\:{x}_{j}$ is the observed value of two points of interest; $\:\stackrel{-}{x}$ is the expected value of x; $\:{w}_{ij}$ is the elements of the spatial weight matrix; and $\:{h}_{0}$ is the normalizing factor, given by $\:{h}_{0}$ =$\:\:\sum\:_{i=1}^{n}\sum\:_{j=1}^{n}{w}_{ij}$. The range of the Moran’s I is [–1, 1], where a value of 1 means that clusters with high child vaccination status values are close to clusters with similar high child immunization status values, while a value of −1 means that high values are close to low values of child immunization status.

### Hot spot and cold spot analysis

It involves computing the Getis-OrdGi* statistic for each feature within a dataset. The resulting z-score and p-value indicate where features with either high or low values cluster spatially. For a feature to be deemed a significant hot spot or cold spot, it should possess a high value and be surrounded by neighboring features with similarly high values or vice versa^45^.

### Spatial interpolation

Spatial interpolation involves the estimation of variable values at un-sampled locations based on sampled locations. It allows for the prediction of values within a specific area using observed data through a method known as interpolation^46,47^. Kriging is widely recognized as the primary technique for spatial interpolation^48^. One unique aspect of kriging methods is their consideration not only of distances between observations but also their intent to capture spatial structure in the data by comparing observations separated by specific spatial distances^39^. For this study ordinary Kriging interpolation was applied to estimate the prevalence of immunization status at non-sampled locations across selected East African administrative regions.

### Spatial ordinal logistic regression model

Spatial Ordinal Logistic Regression is a method that integrates spatial effects into the ordinal logistic regression model^39^. It defines in the following form:

4 $$\:logit\left(p({y}_{i}\le\:j/x)\right)=log\left(\frac{pr\left({y}_{i}\le\:j/x\right)}{pr\left({y}_{i}>j/x\right)}\right)={\theta\:}_{j}+{X}^{{\prime\:}}\beta\:+{\rho\:s}_{i},\:j=\mathrm{1,2},\dots\:,J-1$$

Where: $$\:{\rho\:s}_{i}=\raisebox{1ex}{$\sum\:_{j=1}^{k}{w}_{ij}{\widehat{y}}_{i}$}\!\left/\:\!\raisebox{-1ex}{$\sum\:_{j=1}^{ki}{w}_{ij}$}\right.$$, $$\:{\theta\:}_{j}$$=the threshold value, 𝛽 is a vector of coefficients for explanatory variables X, and ρ is the coefficient of the auto-covariate variable.

## Results

### Exploratory analysis

**Fig. 4:**
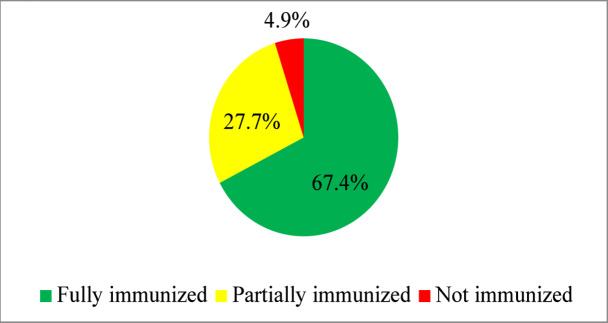
The prevalence of immunization status of children in East Africa.

The analysis included a weighted sample of 22,734 women with children aged 12–23 months. Descriptive statistics, including graphical analysis and chi-square tests, were used to assess the relationship between independent variables and outcome variables. The results indicated that in East Africa, 67.4% of children were fully immunized, and 27.7% of children were partially immunized whereas the remaining 4.9% were not immunized (Fig. 4).

## Spatial analysis

### Spatial distribution of immunization status in East Africa

The distribution of the child immunization status and the covariates suggested wide variations in each of the dependent and independent variables. The mean proportion of the fully, partially, and not immunized rate of the regional communities in East Africa was 51.8%,73.0%, and 12.8% with the working mother’s rate of 73%, and with 36% of the population having media access. 25% of the 132 regions in east Africa had less than 67%, 84%, and 15% of fully, partially, and not immunized children respectively and almost half of the regions had 48%, fully immunized children. 75% of the 132 regions had less than 53% of fully immunized, 73% of partially immunized and 15% of not immunized children. The coefficients of variation for sex of household, working father, media access, wealth index, ANC visits and number of living children were high, showing wide variations among regions in East Africa. Significant autocorrelation was observed for both the child immunization status and most of the independent variables, indicating that the child immunization status and the covariates were highly spatially correlated (Table 1).

**Fig. 5:**
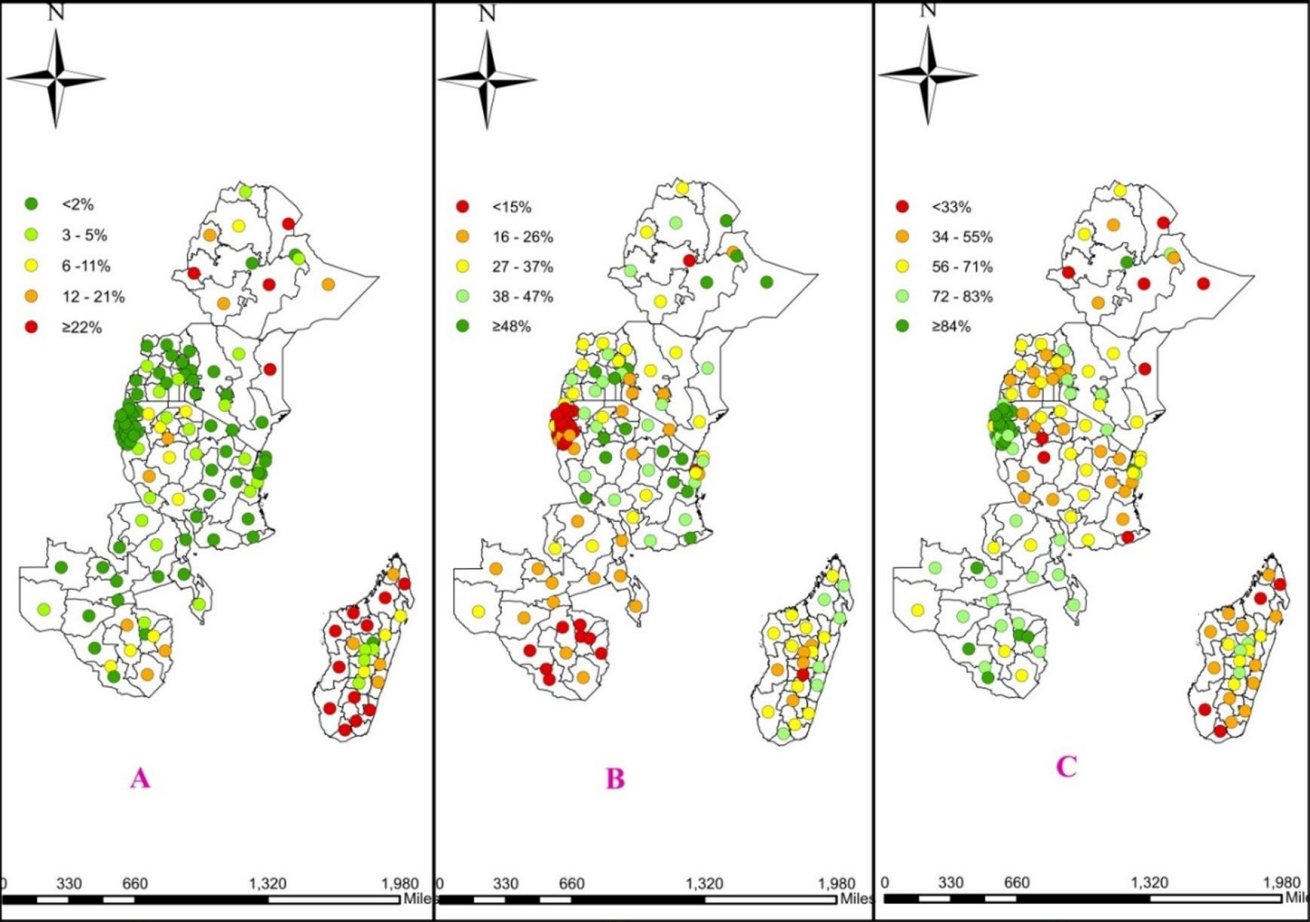
prevalence of (A) Not, (B) Partially, and (C) Fully immunized children across 132 EA regions.

From the total of 132 East African regions, each point in the map characterizes the proportion of not immunized, partially immunized, and fully immunized children of age 12–23 months old. The red color indicates the highest proportion whereas; the green color indicates the lowest proportion for not immunized children and vice versa for the rest categories of the immunization in East African regions (Fig. 5).

**Table 1:** Descriptive statistics of the selected covariates and the moran’s I test statistic.

Variables	Minimum	1 st Quartile	Median	Mean	3rd Quartile	Maximum	SD(CV%)	Moran’s I/Z values
% of fully immunized	43.9	66.7	47.7	51.8	53.0	54.5	5.7 (10.8)	0.028 (2.6) ***
% of partially immunized	39.1	84.3	58.1	73.0	72.5	77.1	6.22 (9.7)	0.004 (3.1) **
% of not immunized	8.56	15.21	14.02	12.77	15.36	19.54	3.28(22.07)	0.34(2.28) ***
Average age of mother	47.6	67.1	42.6	51.2	47.8	51.8	7.1 (15.1)	0.005 (1.9) ***
% of women with illiteracy	49.2	84.3	64.5	69.1	71.2	73.6	5.92 (7.9)	0.144(7.1) ***
% father with literacy	46.7	87.2	64.8	74.1	71.2	68.8	9.13 (11.8)	0.004 (2.45) ***
% of sex of household	7.73	107	60.75	78.23	73.43	110	3.91(32.82)	0.238(5.22) ***
% working women	28.0	83.1	47.1	52.9	52.8	59.6	11.03 (18.5)	0.290 (7.2) ***
% working father	8.9	59.5	28.7	34.6	34.4	41.2	10.35 (27.9)	0.125 (1.7)
% of Media	13.4	58.2	31.8	36.3	37.4	41.0	9.58 (25.8)	0.016 (5.3) ***
% of weight of child	3.6	4.5	3.9	3.9	3.8	4.34	0.26 (5.5)	0.004 (1.4)
% of wealth	0.1	78.3	31.8	39.4	43.2	48.5	15.86 (39.5)	−0.011 (1.8) *
% of children with vitamin A	3.9	49.0	21.4	23.2	25.2	26.2	6.13 (27.5)	0.016 (1.2)
% of rural residence of mother	12.8	32.8	20.3	21.8	22.8	26.4	5.19 (19.5)	0.127 (1.6) *
% of ANC visits	0.8	90.3	33.4	47.2	44.9	64.8	24.7(54.14)	0.434(4.53) ***
% of PNC	23.3	26.4	27.3	28.2	30.1	33.8	3.23(23.58)	0.37(7.62) ***
% of number of living children	0.03	0.42	0.44	0.53	0.71	1.04	0.26(50.12)	1.04(1.78) *
% of Place of delivery	59.2	114.7	78.16	89.8	87.93	97.8	14.70 (14.7)	0.024 (1.72) *
% of health documentation	6.16	9.2	7.2	6.9	7.4	8.4	0.96 (11.2)	−0.034 (11.0) **
% of mother with tetanus injection	10.1	18.3	12.4	12.7	14.2	14.9	1.77 (11.8)	−0.021 (2.2)
% of birth order of child	9.84	43.5	21.2	25.3	25.1	29.2	9.8 (42.2)	−0.013 (1.1)

SD: Standard deviation; CV: Coefficient of variation; *, ** and *** = Moran’s I values are significant at 10%, 5%, and 1% respectively.

### Spatial autocorrelation of immunization status

**Fig. 6:**
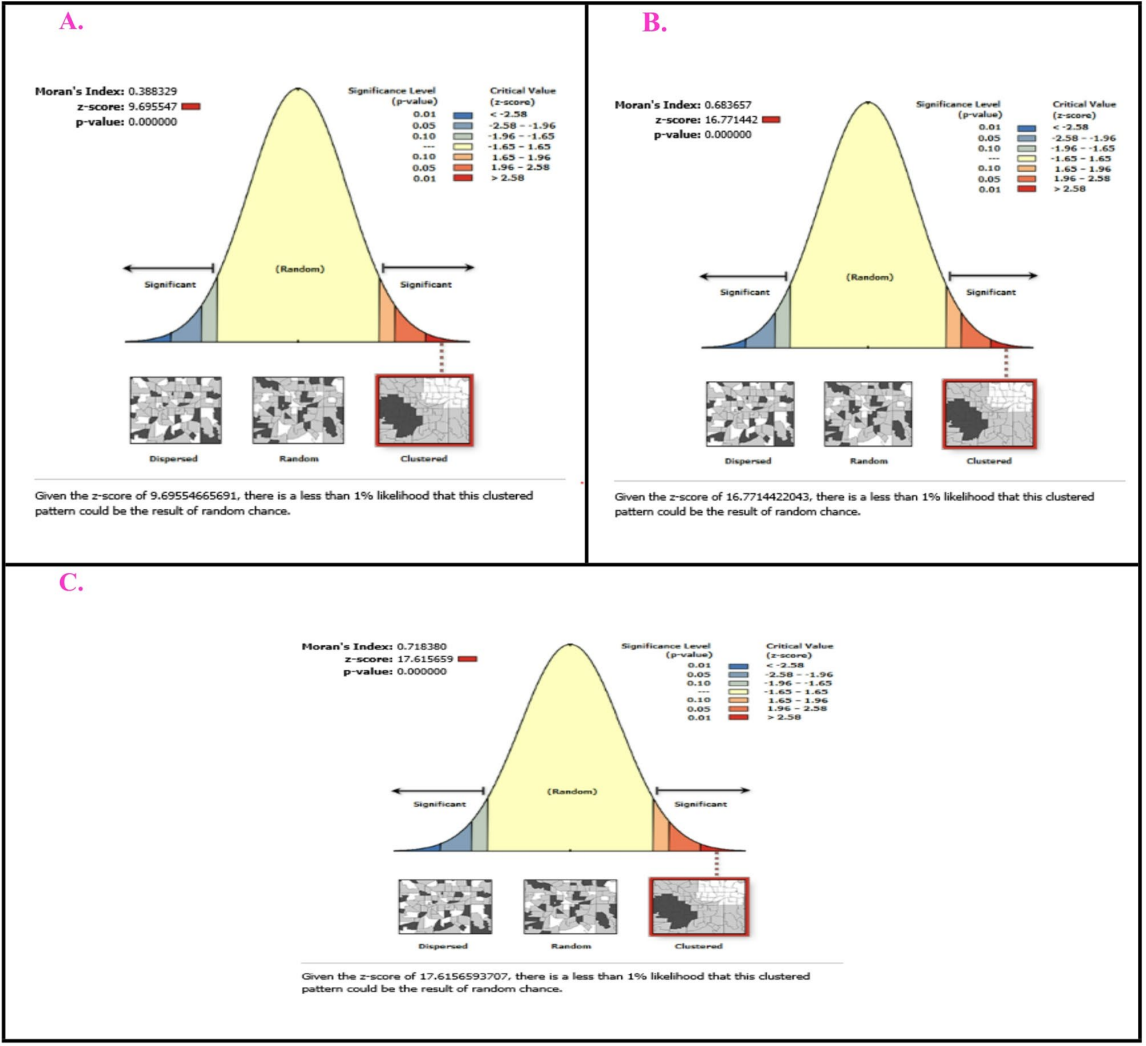
The spatial autocorrelation analysis for (A) Not, (B) Partially, and (C) Fully immunized children in the 132 regions of East Africa.

The estimated Global Moran’s Index of not, partially, and fully immunized children aged 12–23 months were 0.388329, 0.683657 and 0.718380, respectively. When we look at the corresponding Moran’s I p-values all were less than 0.05, then we could conclude that there was significant evidence of spatial autocorrelation in not immunized, partially and fully immunized children in between regions (Fig. 6).

### Hot-Spot analysis of children immunization status in East Africa

**Fig. 7:**
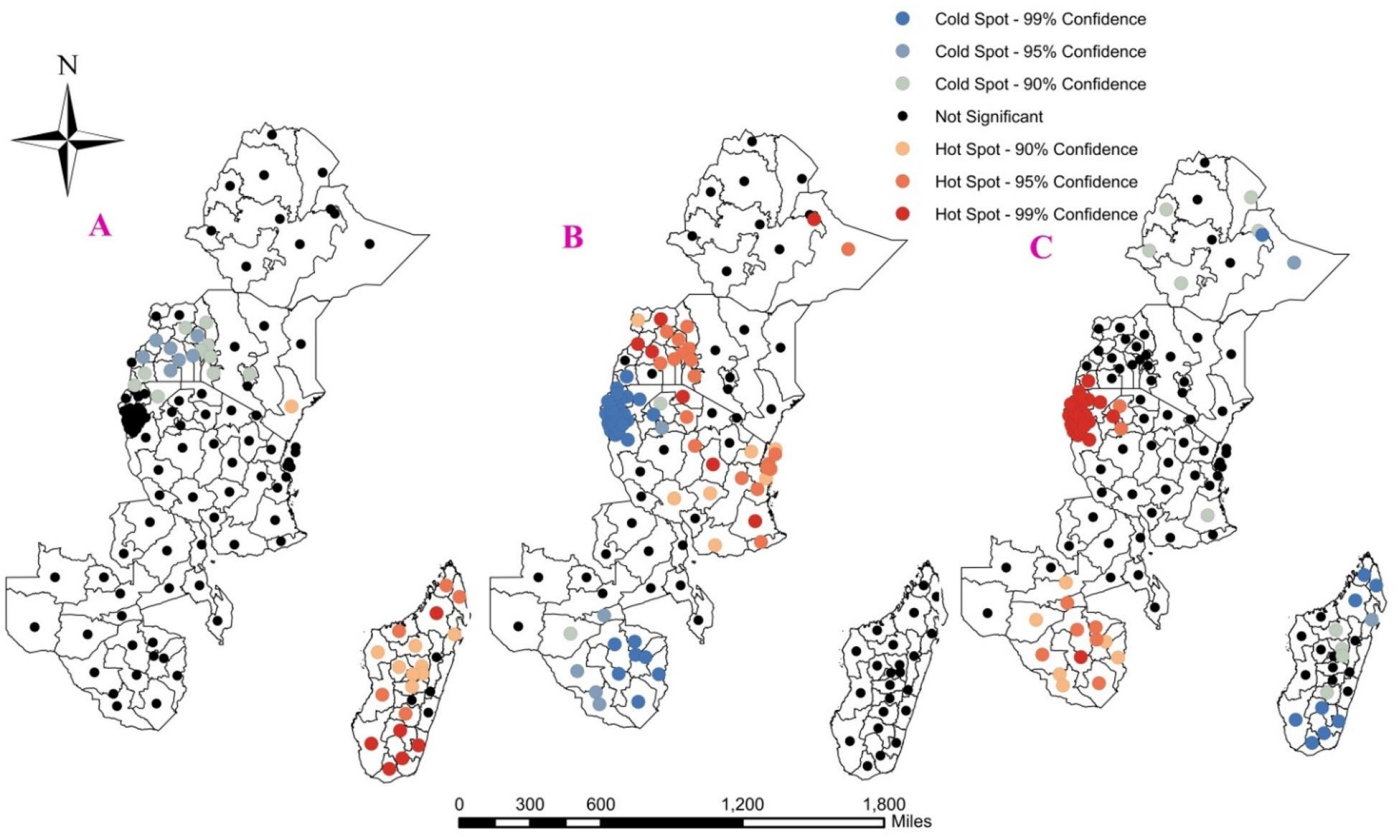
The Hot-spot analysis of (A) Not, (B) Partially, and (C) Fully immunized children’s in EA regions.

The Local Getis-Ord Gi* statistics identified significant high and low coverage areas of immunization status. A point with red color indicates significant high coverage areas, and the blue color indicates the cold spots (low coverage areas) of each category of immunization status. So based on these indicators for not immunized children most parts of Madagascar and coast from Kenya were observed hot spot areas on the other hand, most parts of Uganda, some parts of Rwanda, Central Kenya, and western Kenya were cold spot areas (Fig. 7).

For partially immunized children dire Dawa and Somali from Ethiopia, western Kenya, most regions of Tanzania and Uganda, were hot spot regions while all Burundi and Rwanda regions, and some Zimbabwe regions were cold spot areas.

For fully immunized children all Rwanda and Burundi regions and also some Zimbabwe regions were hot spot areas and most regions in Madagascar were cold spot areas.

### Clusters and outliers of immunization status

The red colors (high-low outlier) indicate a high number of not, partially, and fully immunized children surrounded by the low number of not, partially, and fully immunized children respectively, whereas, the blue colors (low-high outlier) show a low number of not, partially, and fully immunized children surrounded by the high number of not, partially, and fully immunized children respectively.

Based on these indicators Geita were the only regions of Tanzania with high-risk areas surrounded by low-risk areas of not immunized children, while Haute-matsiatra were the only regions of Madagascar in which low risk regions were surrounded by high-risk regions of not immunized children’s.

The regions of Tanzania Kagera and Geita, Ankole from Uganda and Cibitoke from Burundi were regions where the higher number of partially immunized children’s were surrounded by the low number of partially immunized children’s while from regions of Uganda: Bunyoro and Karamajo the regions of Tanzania: Kilimanjaro, Mara, and Sinjida the regions of Kenya: Nyanza and western Kenya were areas in which low number of partially immunized children were surrounded by high number of partially immunized children.

**Fig. 8:**
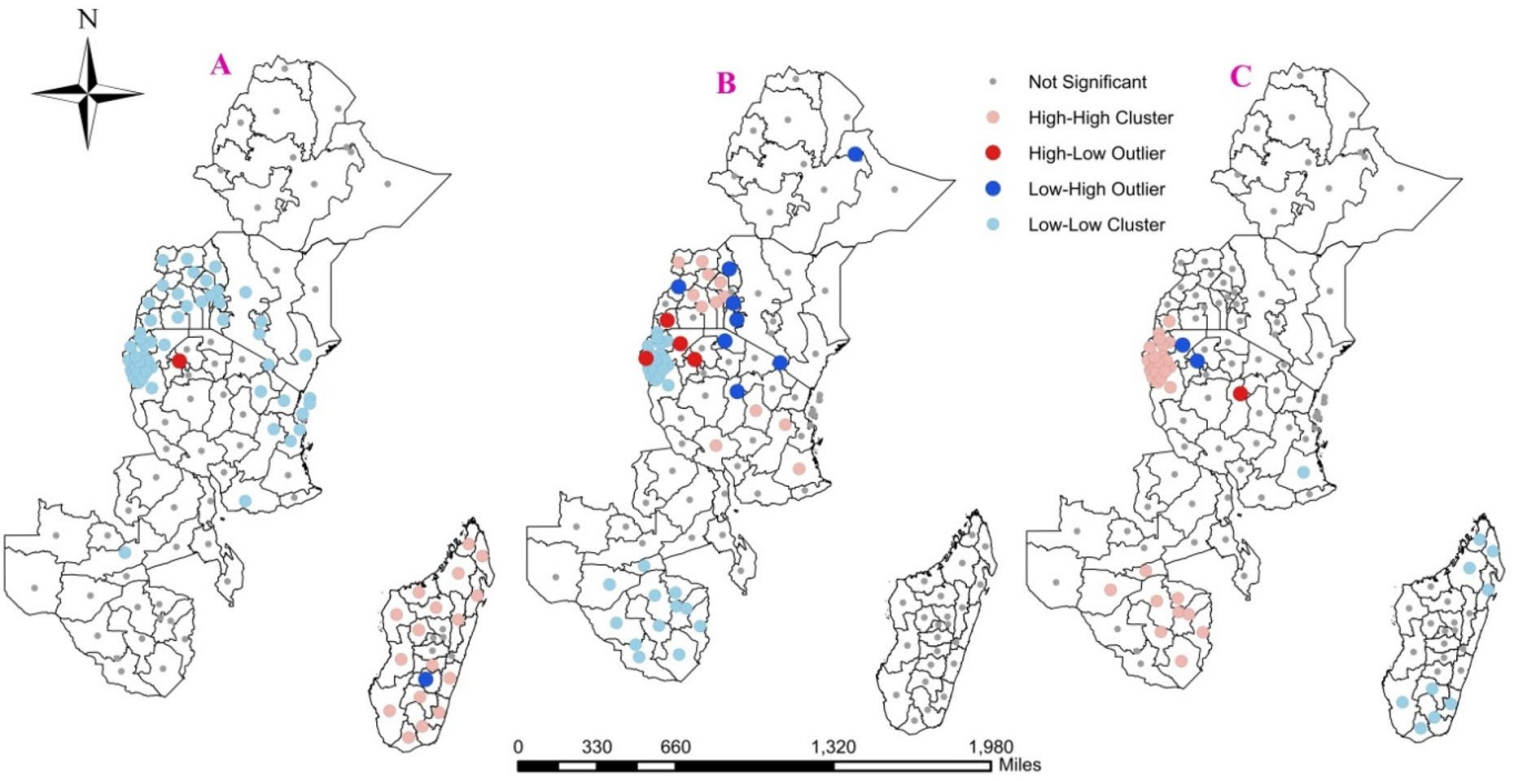
Cluster and Outlier Analysis of (A) Not, (B) Partially, and(C) Fully immunized children’s aged 12–23 months in EA Regions.

Kagera and Geita were the only regions of Tanzania with high frequency of fully immunized children surrounded by low frequency of fully immunized children, on the contrary in this country; Singida was the only region in which low frequency of fully immunized children surrounded by high frequency of fully immunized children (Fig. 8).

High-High cluster (Rose quartz color) means a high proportion of not, partially, and fully immunized children were surrounded by another high proportion neighbors of not, partially, and fully immunized children respectively; Low-Low cluster (Oxide blue color) means a low proportion of not, partially, and fully immunized children were surrounded by another low proportion of neighbors of not, partially, and fully immunized children, respectively.

According to this all regions of Rwanda, Burundi, Uganda, all regions of Kenya except the two eastern and north eastern Kenya, most but not all regions of Tanzania, and central Zambia were regions in which low frequency of not immunized children were found. This means in these regions the proportions of not immunized children were low, and found mostly in neighboring regions. On the other hand, higher numbers of children were clustered in most regions of Madagascar. When we come to partially immunized children Lusaka and southern Zambia, all Zimbabwe regions, Kigoma from Tanzania, all regions of Rwanda and Burundi except Cibitoke were regions in which low proportion of partially immunized children were found in altogether. Also as seen in the descriptive part of our result Rwanda and Burundi, most Zimbabwe regions, south and central Zambia had higher number of fully immunized children, and from this analysis also assure that these children were found in neighboring regions. Also, we evidenced that, fully immunized children were found altogether in most regions of Madagascar (Fig. 8).

### Semi-variogram model of immunization status of children

**Fig. 9:**
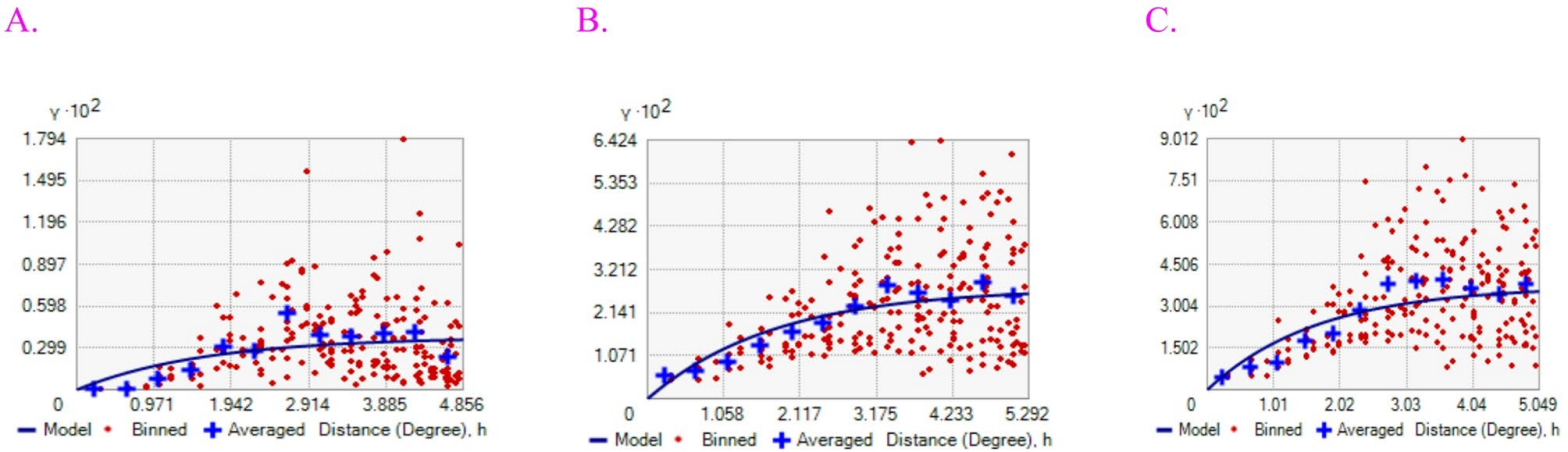
The Semi-variogram graph of (A) Not, (B) Partially, and (C) Fully immunized children’s aged 12–23 months.

The Exponential model was the best for Not, Partial, and Fully immunization children’s with the least value of the mean square error (MSE).The ratio of nuggets to sill expressed in percentages can be regarded as a criterion for classifying. the spatial dependence. If this ratio is less than 25%, then it has strong spatial dependence; if the. ratio is between 25 and 75%, has moderate spatial dependence and greater than 75%, shows weak spatial dependence. The nugget-to-sill ratio for not, partially, and fully immunized children was 0.00784, 0.00763, and 0.00753, respectively. Which tells us the spatial autocorrelations of not, partially, and fully immunized children were 0.78%, 0.76% and 0.75%, respectively, and this represents the degree of strong spatial dependency across East African regions (Fig. 9).

### Spatial interpolation of immunization status among 12–23 months old children

The predictions of higher-prevalence and lower-prevalence areas of not immunized children are indicated by the red and blue colors, respectively. The red color indicates the occurrence of highest number of not immunized children. Whereas for partially and fully immunized children’s vaccination status we use the reverse.

The highest proportion of not immunized children occurred in Tigray regions of Ethiopia and Atsimo-andrefana in Madagascar region. 

**Fig. 10:**
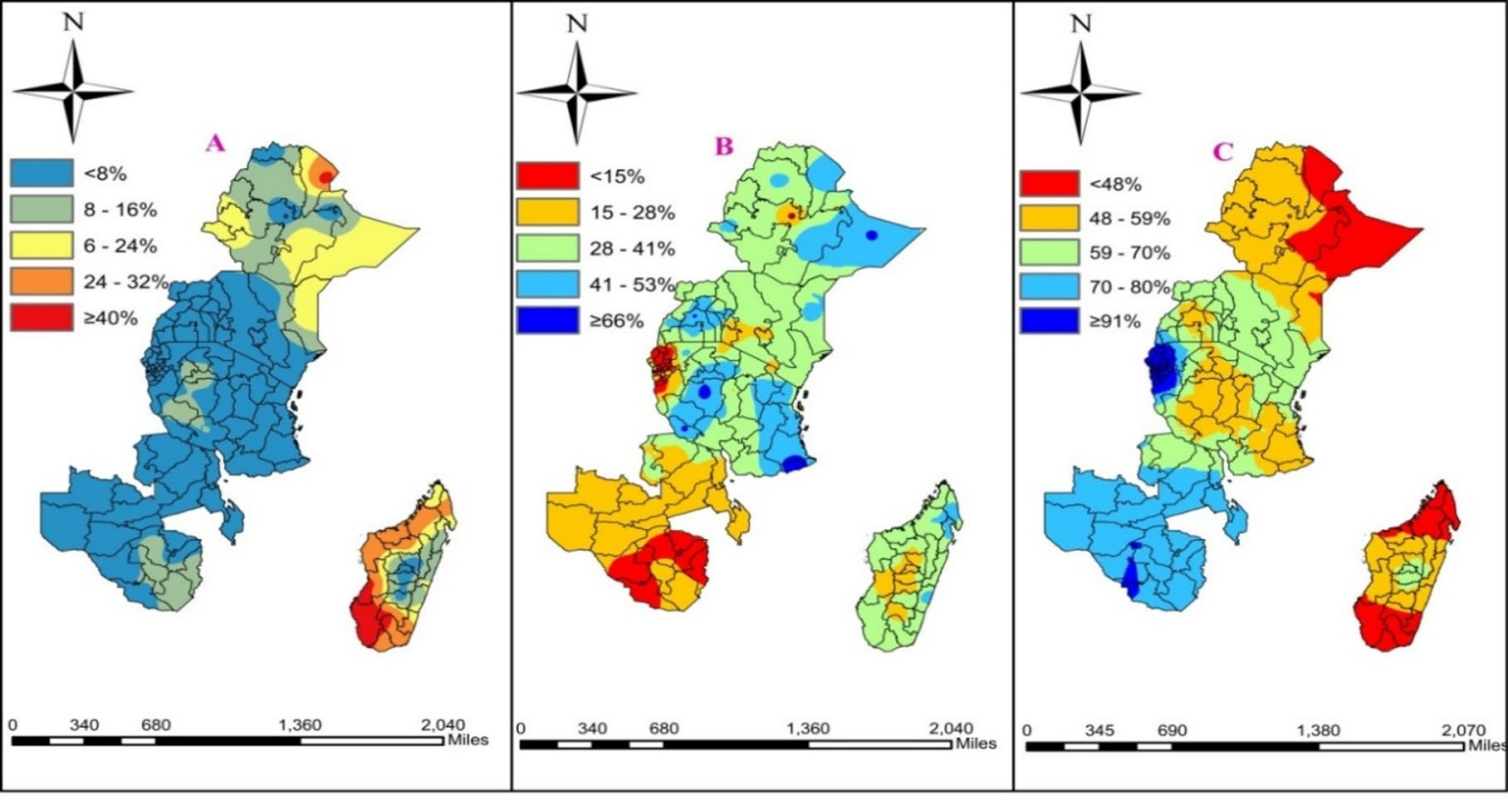
Kriging interpolation of (A) not immunized (B) partially immunized (C) fully immunized children’s.

The highest prevalence of partially immunized children occurred in Somali and Benshangul gumuz regions in Ethiopia, Rukwa, Mtwara, Tabora, Tanga, regions of Tanzania, and north Buganda/central_2_ in Uganda, Tigray regions of Ethiopia and Atsimo-andrefana in Madagascar region. On the other hand, fully immunized children occurred mostly in western Kenya, Copperbelt in Zambia, all regions of Rwanda, and almost all regions of Zimbabwe and Uganda (Fig. 10).

### Ordinal logistic regression analysis

#### Model comparison

The choice between the POM and PPOM was guided by statistical tests. The Score Test for the Proportional Odds Assumption was significant (Chi-Square = 684.36, p-value < 0.0001), indicating a violation of the assumption. Therefore, the standard POM was deemed inappropriate. We then fitted both the PPOM and the more flexible Generalized Ordered Logit Model (GOLM). Model comparison based on Akaike Information Criterion (AIC) and Bayesian Information Criterion (BIC) indicated that the PPOM provided a superior fit to our data (Table 2).

(Table 2). Consequently, the PPOM was selected as the final model for identifying determinants of childhood immunization status. The parameter estimates of the PPOM are presented and interpreted for the significant predictors (at 5% significance level). The parameter estimates from the PPOM are presented and interpreted for predictors that were statistically significant at the 5% level.

**Table 2:** Model comparisons.

Type	AIC	BIC	−2LL	*p*-value
PPOM	25,805.971	26175.78	25713.9711	< 0.000
GOM	25,832.908	26459.976	25676.908	<0.000

### Children immunization status and its associated factors

#### Results of partial proportional odds model

Table 3 presents two panels of results. The first panel compares children who are not immunized with those who are either partially or fully immunized. The second panel contrasts children who are not or only partially immunized with those who are fully immunized from left to the right side of the table. Positive coefficients indicate that higher values of the predictor variable increase the likelihood of being in a higher immunization category, while negative coefficients suggest a greater likelihood of being in a lower category.

From the PPOM results, mother’s education, father’s education, father’s occupation, mother’s occupation, sex of the household head, media exposure, birth interval, child’s weight, health documentation, antenatal care visits, postnatal care visits, taking tetanus vaccination, taking vitamin A1, and country were significantly associated with children’s’ immunization status. Also from this model health documentation, antenatal care visits, postnatal care visits, taking tetanus vaccination, taking vitamin A1, and country doesn’t satisfy the parallel lines assumption. Thus, the model relaxes the constraint of equal coefficients, allowing them to differ between the two equations.

### Predictors satisfying the parallel line assumption

The PPOM results tells us, holding all other variables constant, compared with childrewhose mother had primary, secondary and higher education, children born from uneducated mother have an increased risk of not or partially immunized 0.73 [OR = 0.73, CI: 0.67 0.80), 0.58 [OR = 0.58, CI: 0.52 0.65], and 0.55 = (OR = 0.55; CI: 0.45 0.68) respectively. The odds of not or partially immunized children was 0.85, 0.84 and 0.78 times lower among children born from father who had primary, secondary and higher education respectively as compared to children born from uneducated father. Regarding with occupational status, employed father were 0.85 (OR = 0.85, CI: 0.76 0.95) times less likely to be not or partially immunized their children than that unemployed father. Similarly, children who were born from employed mothers were 0.90 (OR = 0.90, CI: 0.84 0.97) times less likely to be not or partially immunized their children than those children who were from house wife mother.

In comparison to male headed households, the fitted model had showed that children from female headed households were 1.1 (p-value = 0.0056) times more likely to be not or partially immunized. Children living in families with access of media exposure were 0.87(OR = 0.87, CI: 0.81 0.93) times less likely to be not or partially immunized as compared to those children living in families with no access of media exposure. As compared to children from families with birth interval of 24–47 months and above 47 months, the odds of being in a not or partially immunized status were 0.86 (OR = 0.86, p-value = 0.0015) and 0.78 (OR = 0.78, p-value = 0.0001) times lower for children from families with birth interval of < 24 months. Being not or partially immunized were 0.92 (OR = 0.92, CI: 0.85 0.99) times lower for children who have average birth weight as compared to small birth weight (Table 3).

### Predictors that do not satisfy the parallel regression assumption

The children from families with health documentation were found 0.04 (OR = 0.04; CI: 0.03 0.04) times less likely to be in a not immunized status rather than in a partially or a fully immunized status when compared to children from families with no health documentation. In contrast to children from families with no health documentation, children from families with health documentation were 0.12 (OR = 0.12; CI: 0.11 0.13) times less likely to be in a not or partially immunized status rather than in a fully immunization status.

The estimated odds of children being not immunized, rather than being partially or fully immunized, were 0.34 (OR = 0.34, CI: 0.28 0.42) times higher among those whose mothers had antenatal care visit, compared to children whose mothers had no antenatal care visit. Also, compared to those whose mothers had no antenatal care visit, the odds of not or partially immunized children for mothers who had ANC visit were decreased by 47.7% [OR = 0.52, CI: 0.45 0.61]. The estimated odds of being not immunized compared to being partially or fully immunized were 0.78 [OR = 0.78, p-value = 0.001] times higher among those whose mothers had postnatal care follow up, compared to mothers who did not have postnatal care follow up a child born to mothers who had 1–2, and 3 + tetanus injections was 0.64 (OR = 0.64, CI: −0.53 0.78) and 0.71 (OR = 0.71 CI: 0.56 0.91) times less likely of being not immunized than a child born to mother who did not had received tetanus injections, respectively (Table 3).

The estimated odds of children being not immunized, compared to being partially or fully immunized were 0.36 times lower among those whose mothers had received vitamin A_1,_ compared to children whose mothers didn’t received vitamin A1. The estimated odds of children whose mothers had received vitamin A_1_ being not or partially immunized were lower by 52% of the estimated odds of children whose mothers did not received vitamin A_1_.

Regarding with country, the results of PPOM showed that compared to a child from Burundi and Uganda, a child from Ethiopia was 0.30 (OR = 0.30, CI: 0.17 0.52), and 0.54 (OR = 0.54, CI: 0.36 0.81), times less likely to be in a not immunized rather than in a partially or a fully immunized status. Compared to a child in Madagascar, Tanzania and Zimbabuwe, a child from Ethiopia were 5.30 (OR = 0.30, CI: 4.23 6.62), 2.00 (OR = 2.00, CI: 1.48 2.72), 6.87 times (OR = 6.87, CI: 4.91 9.60), times more likely to be in a not immunized rather than in a partially or a fully immunized status. In Contrast to a child from Ethiopia, a child from Burundi and Rwanda were 0.40 (p-value = 0.0001) and 0.22 (p-value = 0.0001) times less likely to be in a not or partial immunization status rather than in a full immunization status. Children from countries of Kenya, Madagascar, Tanzania, and Uganda were found 1.52 (OR = 1.52, CI: 1.30 1.77), 1.98 (OR = 1.98, CI:1.69 2.34), 2.33 (OR = 2.33, CI: 1.98 2.73) and 2.41 (OR = 2.41, CI: 2.06 2.82) times more likely to be in not or partially immunized rather than in a fully immunized status when compared to children Ethiopia.

**Table 3:** Maximum likelihood estimates of partial proportional odds model.

Predictors	Categories	Not immunized Vs partially or fully immunized			Not or Partially immunized Vs fully immunized		
		Estimate	S.E	AOR(95%CI)	Estimate	S.E	AOR(95%CI)
Intercept	-	0.639	0.106	1.895(1.501, 2.289)	2.803	0.108	16.495 (13.003, 19.987)^***^
Mother education level	No education			1			1
	Primary	−0.314	0.046	0.730 (0.668, 0.799) ^***^	−0.314	0.046	0.730 (0.668, 0.799) ^***^
	Secondary	−0.550	0.057	0.577 (0.516, 0.645) ^***^	−0.550	0.057	0.577 (0.516, 0.645) ^***^
	Higher	−0.596	0.104	0.551 (0.450, 0.675) ^***^	−0.596	0.104	0.551 (0.450, 0.675) ^***^
Father education level	No education			1			1
	Primary	−0.162	0.047	0.851 (0.775, 0.933) ^***^	−0.162	0.047	0.851 (0.775, 0.933) ^***^
	Secondary	−0.177	0.056	0.838 (0.751, 0.934) ^***^	−0.177	0.056	0.838 (0.751, 0.934) ^***^
	Higher	−0.244	0.083	0.783 (0.666, 0.921) ^***^	−0.244	0.083	0.783 (0.666, 0.921) ^***^
Occupation of father	No			1			1
	Yes	−0.165	0.057	0.848 (0.758, 0.949) ^***^	−0.165	0.057	0.848 (0.758, 0.949) ^***^
Occupation Mother	House wife			1			1
	Employed	−0.104	0.038	0.901 (0.837, 0.970) ^***^	−0.104	0.038	0.901 (0.837, 0.970) ^***^
Sex of head of housed hold	Male			1			1
	Female	0.091	0.038	1.095 (1.015, 1.180) ^***^	0.091	0.038	1.095 (1.015, 1.180) ^**^
Media exposure	No			1			1
	Yes	−0.138	0.035	0.871 (0.813, 0.934) ^***^	−0.138	0.035	0.871 (0.813, 0.934) ^***^
Birth interval	< 24 months			1			1
	24–47 months	−0.153	0.048	0.858 (0.781, 0.943)^***^	−0.153	0.048	0.858 (0.781 0.943)^***^
	>=48 months	−0.252	0.052	0.777 (0.701 0.861)^***^	−0.252	0.052	0.777 (0.701 0.861)^***^
Weight of child	Small			1			1
	Average	−0.089	0.038	0.915 (0.850 0.985)^**^	−0.089	0.038	0.915 (0.850 0.985)^**^
	Large	0.061	0.049	1.063 (0.967 1.169)	0.061	0.0485	1.063 (0.967 1.169)
U_Health documentation	No			1			1
	Yes	−3.356	0.113	0.035 (0.028 0.043)^***^	−2.123	0.042	0.120(0.110 0.130)^***^
U_Antenatal care follow up	No			1			1
	Yes	−1.082	0.106	0.339 (0.275 0.418)^***^	−0.648	0.080	0.523(0.447 0.612)^***^
U_Postnatal care follow up	No			1			1
	Yes	−0.244	0.097	0.783 (0.648 0.947)^**^	−0.045	0.040	0.956 (0.884 1.034)
U_Tetanus injection before birth	Not received			1			1
	1–2	−0.445	0.098	0.641 (0.529 0.776)^***^	−0.068	0.044	0.934 (0.856 1.019)
	3 and above	−0.341	0.123	0.711 (0.559 0.905)^***^	−0.026	0.056	0.974 (0.873 1.087)
U_Talking vitamin A1	No			1			1
	Yes	−1.031	0.081	0.357 (0.305 0.418)^***^	−0.723	0.039	0.485 (0.450 0.524)^***^
U_Country	Ethiopia			1			1
	Burundi Kenya Madagascar Malawi Rwanda Tanzania Uganda Zambia Zimbabuwe	−1.207 0.075 1.667 −0.058 −0.273 0.695 −0.613 −0.339 1.927	0.283 0.135 0.114 0.183 0.406 0.155 0.206 0.231 0.171	0.299 (0.172 0.521)^***^ 1.077(0.827 1.404) 5.295 (4.233 6.624)^***^ 0.944 (0.660 1.351) 0.761 (0.344 1.685) 2.003 (1.478 2.716)^***^ 0.542 (0.362 0.811)^***^ 0.712 (0.453 1.119) 6.867 (4.912 9.599)^***^	−0.929 0.416 0.685 −0.143 −1.535 0.845 0.879 0.048 −0.015	0.089 0.078 0.083 0.083 0.142 0.082 0.081 0.092 0.114	0.395 (0.332 0.470)^***^ 1.516(1.300 1.767)^***^ 1.984(1.687 2.335)^***^ 0.867 (0.737 1.019) 0.215 (0.163 0.285)^***^ 2.328 (1.984 2.733)^***^ 2.407 (2.056 2.819)^***^ 1.049 (0.876 1.257) 0.985 (0.788 1.233)

Key: ** and *** Significant at 0.01 and 0.001 level of significance, S.E = standard Error, CI = Confidence interval, AOR = Adjusted Odds Ratio, 1 = estimate value for reference group, and U_ indicates variables that do not satisfy proportionality assumption.

## Discussion

This study aimed to identify the factors influencing childhood immunization status among children aged 12 to 23 months across 10 East African countries. It also sought to map potential spatial patterns of immunization in relation to regional health service institutional structures. Notably, this is the first study in the region to introduce new measures for assessing childhood immunization prevalence by using aggregated immunization indicators and applying them to large, nationally representative datasets. We used the latest demographic data. This might help stakeholders to plan specific interventions in the provinces in a certain country since there is a diverse experience among provinces in each country. The study findings suggest a clear spatial pattern of child immunization status across the administrative regions of East Africa. The Moran’s I statistics suggested the measure of spatial dependence among the outcome and the risk factors. Moreover, it was found to be highest father education level, sex of head household, mother employment status, media exposure of parents, ANC, PNC, health documentation and the like. The predicted values revealed that a high level of not immunized children was found in certain administrative regions of Ethiopia and across most regions of Madagascar^13,14,49^.

The proportion of children that experienced fully, partially, and not immunized status was 67.4, 27.7, and 4.9%, respectively. According to prior studies, this is the highest figure (i.e. fully immunized) conducted a decade ago^50^.

The partial proportional odds model has demonstrated a significant positive association between the outcome variable (childhood immunization status) and the child, maternal, environmental, and house hold related characteristics. The results, consistent with the studies reported in East Africa^11^ regions, revealed that maternal education as potential determinant factor of child immunization status. Compared with a child with formal maternal education, a child with no maternal education has more risk of being in a worse immunization status. Several previous studies from SSA, East Africa, Ethiopia, Bangladesh, Tanzania and Uganda complied with this finding supporting the notion that children whose mothers have formal education were less likely to be not immunized as compared with children whose mother had no education^9,11–14,49–52^ indicating that maternal education is an essential factor affecting infant immunization practices. As educated mothers have better knowledge of child health, they are more conscious of their child’s health and look after their children better^4^. Not only maternal education but also father’s education significantly associated with childhood vaccination status, and children whose father attended formal education had less chance of being not immunized. Other studies reported similar findings^9^. Fathers with formal education know better about the advantage of childhood immunization, which contribute positively to preventing childhood fatal disease.

Of course, it is also important to consider the employment status of the parents in this respect. With more than 5.2% of women in East Africa being unemployed and usually spend more of their time with their children, they would afford to provide childcare and vaccination services as compared to employed women could do. Thus, while the level of education significantly contributes to the mothers’ capacity for child care and vaccination, the time factor might have complemented this impact^9,53^.

Our study further found that the odds of children not being immunized are higher in female-headed households compared to male-headed ones^53^. This is because Women heading households often juggle multiple responsibilities, leaving little time for routine health services like vaccinations. Additionally, structural barriers such as provider bias or limited female-friendly outreach may further hinder access.

Apart from house hold related determinants the parents’ access to mass media use also impacted the immunization status of the children. As such, a significant positive association is observed between the two variables, suggesting that parents who are exposed to media learn about the health of their children. This finding is consistent with previous research evidence reported in other countries^11,14,22^.

Another key determinant was the child’s birth interval, which showed a negative association with immunization status. Shorter birth spacing may lead to increased mental and psychological stress for mothers, potentially reducing their likelihood of utilizing childhood vaccination services. This is consistent with the studies done by^11,14^. This work suggests weight of child at birth has significant association with child vaccination which is consistent with a similar study in East Africa^14^.

Moreover, the likelihood of children not being immunized and the likelihood of children being not or partially immunized was higher among those whose mothers lacked health documentation, did not take Vitamin A1, had no antenatal (ANC) or postnatal (PNC) care visits, and did not receive tetanus toxoid (TT) injections during pregnancy^12,14,16,22,49^. The possible reasons may be that these factors reflect limited maternal engagement with healthcare services, which can negatively impact child immunization. Mothers without health documentation may lack essential records to track vaccination schedules, increasing the risk of missed doses. Not taking Vitamin A1 and not receiving TT injections during pregnancy may indicate poor access to or underutilization of maternal health services also causes child immunization. Similarly, the absence of antenatal (ANC) and postnatal care (PNC) visits reduces opportunities for mothers to receive information, counseling, and reminders about the importance of vaccinating their children. Collectively, these gaps contribute to lower vaccination coverage among their children.

A further look into the results shows that, country is a significant factor of child immunization status^9,11,14^. A child living in Ethiopia has a higher risk of not immunized compared to a child in Burundi and Uganda, while compared to a child in Madagascar, Tanzania and Zimbabwe; it has lower risk of not immunized. Also a child living in Ethiopia has a higher risk of not or partially immunized compared to a child in Burundi and Rwanda, while compared to a child in Kenya, Madagascar, Tanzania and Uganda; it has lower risk of not or partially immunized^49^. This is likely due to variations in healthcare systems, policy implementation, and sociocultural contexts across nations. Differences in data systems and record-keeping can also influence measured outcomes.

## Conclusion

The study findings help in theorizing the relationship between the regional administrative level of child immunization status and its determinants. This paper employed a spatial statistical analysis that helps to identify the regional/province level variations of potential covariates that facilitate the child immunization disparities and distributions. The global Moran’s I test confirmed the presence of spatial dependence of child immunization. The result of the analysis justifies the use of spatial data exploration for both the covariates and the outcome variables for identifying population of clusters with high risk of not immunized children, partially immunized children and clusters of fully immunized children. Hence, maximizing basic childhood vaccination in East Africa would be a realistic WHO target, when the concerted interventions are made to minimize barriers at the regional administrative area particularly in these disadvantaged high focus regions. Moreover, different ordinal logistic regression models were formulated in this study and the results confirmed that incorporating the spatial effect in the model gives a better result than that of the traditional ordinal regression model. The model selection criteria revealed that the PPOM model gives the best output over the remaining models in this study. The spatial analyses suggested a statistically significant association of child immunization with women’s literacy rate, the sex of household head, working status of the mother, media exposure of parents, ANC, PNC, and health documentation. To alleviate the problem of low child immunization, the decision makers would focus on those significant covariates. Although our model incorporated spatial effects at the regional level, it did not account for the hierarchical data structure (children nested within households and clusters). Therefore, future research would benefit from a multilevel or survey-adjusted ordinal model with random intercepts for countries. Such a model could further partition the variance attributable to country-level heterogeneity and offer additional insights.

## Data Availability

Publicly available datasets were analyzed in this study. This data can be found at: https://www.dhsprogram.com/Data.
